# A novel gear RUL prediction method by diffusion model generation health index and attention guided multi-hierarchy LSTM

**DOI:** 10.1038/s41598-024-52151-y

**Published:** 2024-01-20

**Authors:** Xinping Chen

**Affiliations:** College of Artificial Intelligence and Big Data, Chongqing College of Electronic Engineering, Chongqing, 401331 China

**Keywords:** Mechanical engineering, Electrical and electronic engineering

## Abstract

Gears, as indispensable components of machinery, demand accurate prediction of their Remaining Useful Life (RUL). To enhance the utilization of ordered information within time series data and elevate RUL prediction precision, this study introduces the attention-guided multi-hierarchy LSTM (AGMLSTM). This innovative approach leverages attention mechanisms to capture the intricate interplay between high and low hierarchical features of the input data, marking the first application of such a technique in gear RUL prediction. Additionally, a refined health indicator (HI) is introduced, constructed through a diffusion model, to precisely reflect the gears' health condition. The proposed RUL prediction method unfolds as follows: firstly, HIs are computed from gear vibration data. Subsequently, leveraging the known HIs, AGMLSTM predicts future HIs, and the RUL of the gear is determined upon surpassing the failure threshold. Quantitative analysis of experimental results conclusively demonstrates the superiority of the proposed RUL prediction method over existing approaches for gear RUL estimation.

## Introduction

Recently, with the rapid development of Industry 4.0, engineering equipment has become increasingly complex and intelligent. In practice, the reliability and stability of equipment operation is an important prerequisite for completing the preset tasks, therefore extremely rigorous requirements are put forward. Given that the gear is the critical and costly drivetrain component in wind turbines and aero-engines, whose failure makes downtime, operations, and maintenance (OM) costs, and, to some extent, casualties^1,2^. Thus researchers pay more attention to the approaches to remaining useful life (RUL) prediction^3–5^. RUL refers to the expected continuous normal working time of the gear from the present to the occurrence of potential failures^5^. The RUL prediction, as an important role in prognostics and health management (PHM), enables the predicted OM decision assistance, which helps ensure equipment stability and avoid damage.

After continuous exploration and verification, the prediction of RUL has yielded significant theoretical research results in the academic world and holds vast potential for application in the industrial sector. RUL prediction approaches are broadly categorized into three classes: method-based^6,7^, data-driven^3,4^, and hybrid^8,9^. These methods exhibit distinct characteristics, but with the rapid advancement of technologies like artificial intelligence^5^, sensor technology^10^, and signal processing technology^11^, the data-driven approach has emerged as the mainstream method for RUL prediction, particularly in complex engineering equipment. Besides data-driven method is more convenient than the model-based method and hybrid method which require a certain expert knowledge of failure mechanism. These characteristics enable data-driven methods suitable for RUL prediction and become a research hot.

Deep learning (DL), being the most popular method in the data-driven approach, has shown remarkable success in machine PHM^12^. Recently, numerous RUL prediction methods based on DL have been proposed by scholars. Ren et al.^3^ introduced a simple DL method for machine RUL prediction, incorporating features in the time domain and frequency domain into a fully connection NN. Meanwhile, Yang et al.^4^ developed a novel DL method with the first convolution neural network (CNN) as the detector of the initial failure point of rotating machinery, and the second CNN as the RUL predictor. Cheng et al.^5^ first designed the CNN to construct the health indicator from the raw data pre-processed by the Hilbert-Huang Transform (HTT), then estimated the machine RUL by SVR regression.

Long short-term memory (LSTM)^13^ as the famous recurrent neural network (RNN) variant not only has its recursive properties but also has unique gating mechanisms, which makes it very suitable for processing sequential data compared with other neural networks (NNs). Therefore, LSTM as a RUL predictor is becoming more and more popular in PHM field. Yang et al.^14^ executed mounts of experiments to find the advantage of the operation information data in the improvement of RUL prediction by using the LSTM models. Wu et al.^15^ proposed a deep LSTM model to estimate bearing RUL via multiple sensor signals. Yuan et al.^16^ investigated the prognostic performance of several RNNs for RUL estimation of aero-engines, including normal RNN, gated recurrent unit (GRU), and LSTM. Wang et al.^17^ presented a novel RUL prediction approach. Firstly, the bearing degradation curve was classified into multiple stages, and then the RUL was obtained by multi-step prediction according to the stage. For the joint tasks of fault assessment and RUL estimation, Miao et al.^18^ designed and established a dual-learning LSTM model. Chen al.^19^ adopted an attention mechanism to weigh the data of different time steps in the cellular to improve the predictive ability of the improved LSTM. Qin al.^20^ proposed a novel attention mechanism to screen the important information before and after inputting the hidden layer of GRU and further improve the roll bearing's RUL prediction accuracy. The above methods improve the LSTM from multiple angles, e.g. combined with CNN feature extraction, artificial feature construction, attention mechanism selection, and other techniques, to obtain better prediction performance. On the other side, researchers find that there is another feature named ordered information hidden in the sequence information which is helpful for RUL estimation, and LSTM based on ordered information (On-LSTM) is firstly proposed to deal with the feature and applied on gear RUL prediction in literature^21,22^. Based on the angle, literatures^23,24^ further explored the usage of ordered information on RUL pre-diction tasks by using attention-guided and mining the mixed zone of hierarchies. The studies about gear RUL prediction have been developed and applied. However, there still exist two main gaps in the methods of gear RUL prediction.

1. One is that the prediction method can not mine ordered information of HIs fully and reasonably, which can decrease the feature extraction ability of models and impact the RUL prediction accuracy.
2. Another is that there is rare work on the construction of HI with clear degradation trends and stable failure theories.

Facing the challenge, the article proposed a novel attention-guided multi-hierarchy LSTM (AGMLSTM) model. AGMLSTM not only can mine the feature of mixed hierarchy but also has the ability which is guided by the attention mechanism reasonably. Thus AGMLSTM is more suitable for gear RUL prediction. Besides, a suitable health index (HI) is beneficial for RUL prediction accuracy. In the paper, a novel HI which is smooth and has a clear trend constructed by the diffusion model is presented. Finally, based on the known HIs, the AGMLSTM is used to predict the future HIs step by step until it exceeds the preset failure value, and the RUL of gear is finally obtained. The outperformance of the presented RUL approach is illustrated by the quantitative evaluation of various indexes during the experiments. Particularly noteworthy is the remarkable achievement of 92% RUL prediction accuracy in the challenging task of predicting gear RUL within one hour, signifying the practical significance of our approach in online RUL prediction.

The main contributions in the article are as follows:

1. The adoption of the diffusion model represents a pioneering approach to constructing the HIs for gears, effectively mitigating fluctuations. Gear HI curves exhibit declining trends, and their failure thresholds are similar.
2. AGMLSTM is proposed for gear RUL prediction. This method demonstrates enhanced capability in extracting ordered information, improving feature extraction, and boosting RUL estimation.
3. Building on the diffusion model and AGMLSTM, the study proposes a novel prediction method, validated through comprehensive assessments of full-life vibration data for gears."

The remainder of the article is arranged as follows. “Theoretical basis” not only introduces the concept of diffusion model but also introduces LSTM. The details of the proposed methods are described in “The proposed methodology”. The experiments with results analysis are given in “Experimental analysis”. Last, in “Conclusion”, the conclusion is summarized.

## Theoretical basis

## Diffusion model

**Figure 1:**
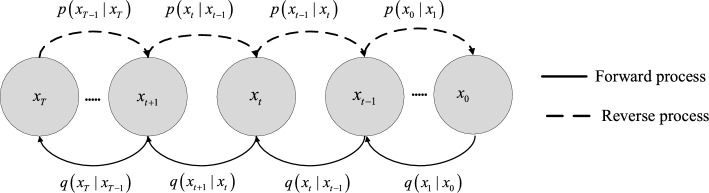
The details of diffusion model.

Diffusion model^25^ is a novel advanced deep generative model. It gradually transforms data into noise and then learns the de-noising process to generate new samples in both forward and backward directions. Thus The learned de-noising module of diffusion model is adopted to construct gear HIs. Figure 1 illustrates the intuition behind the Diffusion model.

In this study, the de-noising diffusion probabilistic model is employed, which operates through the utilization of two Markov chains. Diffusion Model adopts a progressive nosing and de-noising approach. In the forward process, Gaussian noise is gradually added to the original data layer by layer until it transforms into a simple prior Gaussian distribution. In the reverse process, the noise is gradually eliminated by the deep neural network. The fixed approximate posterior $q\left( {x_{1:N} |x_{0} } \right)$ in the forward stage is calculated in Eqs. (1) and (2),

1 $$ q\left( {x_{1:N} |x_{0} } \right) = \prod_{n = 1}^{N} q\left( {x_{n} |x_{n - 1} } \right) $$

2 $$ q\left( {x_{n} |x_{n - 1} } \right)\sim N\left( {x_{n} ;\sqrt {1 - \beta_{n} } x_{n - 1} ,\beta_{n} I} \right) $$

where $\beta_{n} \in \left( {0,1} \right)$, *N* and* I* are the added Gaussian noise, sample number, and identity matrix. While at the reverse process, a learnable Gaussian transition which is beginning at $p\left( {x_{n} } \right)$, with another Markov chain constructs the joint distribution $p_{\theta } \left( {x_{n - 1} |x_{n} } \right)$, as calculated in Eqs. (3) and (4),

3 $$ p_{\theta } \left( {x_{0:N} } \right) = P\left( {x_{N} } \right)\prod\limits_{n = 1}^{N} {p\left( {x_{n - 1} |x_{n} } \right)} $$

4 $$ p_{\theta } \left( {x_{n - 1} |x_{n} } \right)\sim N\left( {x_{n - 1} ,\mu_{\theta } \left( {x_{n} ,n} \right),\delta_{\theta } \left( {x_{n} ,n} \right)I} \right) $$

where mean $\mu_{\theta }$ and variance $\delta_{\theta }$ are obtained from a deep NN.

The objective of the reverse Markov chain, i.e., computing $p_{\theta } \left( {x_{n - 1} |x_{n} } \right)$, is to remove the Gaussian noise introduced during the forward process. The de-nosing object is $p_{\theta } \left( {x_{n - 1} |x_{n} } \right)$ for the reverse Markov chain. Supposed that x_0_ is sampled from the noise $p\left( {x_{n} } \right)$, repeating the process from $p_{\theta } \left( {x_{n - 1} |x_{n} } \right)$ until $n = 1$.

For accurate sampling, make the trained reverse Markov chain $p_{\theta } \left( {x_{n - 1} |x_{n} } \right)$ close to the posterior distribution $q\left( {x_{n - 1} |x_{n} ,x_{0} } \right)$ of the forward process given $x_{0}$. And Kullback–Leibler (KL) divergence is chosen as the similarity evaluation metric, whose equations are defined as bellows,

5 $$ \begin{gathered} D_{KL} \left( {q\left( {x_{n - 1} |x_{n} ,x_{0} } \right)||p_{\theta } \left( {x_{n} - 1|x_{n} } \right)} \right) \hfill \\ = E_{q} \left[ {\frac{1}{2\sum \theta }||\tilde{\mu }_{n} \left( {x_{n} ,x_{0} } \right) - \mu_{\theta } \left( {x_{n} ,n} \right)||^{2} } \right] + C \hfill \\ \end{gathered} $$

In the equation, *C* is a constant that is independent of θ and $\tilde{\mu }_{n}$ represents the average value of $q\left( {x_{n - 1} |x_{n} ,x_{0} } \right)$. And the simplified objection is calculated in Eq. (6) by adding the noise NN $\varepsilon_{\theta }$ with parameters θ,

6 $$ E_{{x_{{0\sim q\left( {x_{0} } \right)}} ,\varepsilon \sim N\left( {0,I} \right)}} = \left[ {\lambda \left( n \right)||\varepsilon - \varepsilon_{\theta } \left( {\sqrt {\overline{\alpha }_{n} } x_{0} + \sqrt {1 - \overline{\alpha }_{n} } \varepsilon ,n} \right)||^{2} } \right] $$

where $\lambda \left( n \right)$ is the function of positive weight.

### Long short term memory

**Figure 2:**
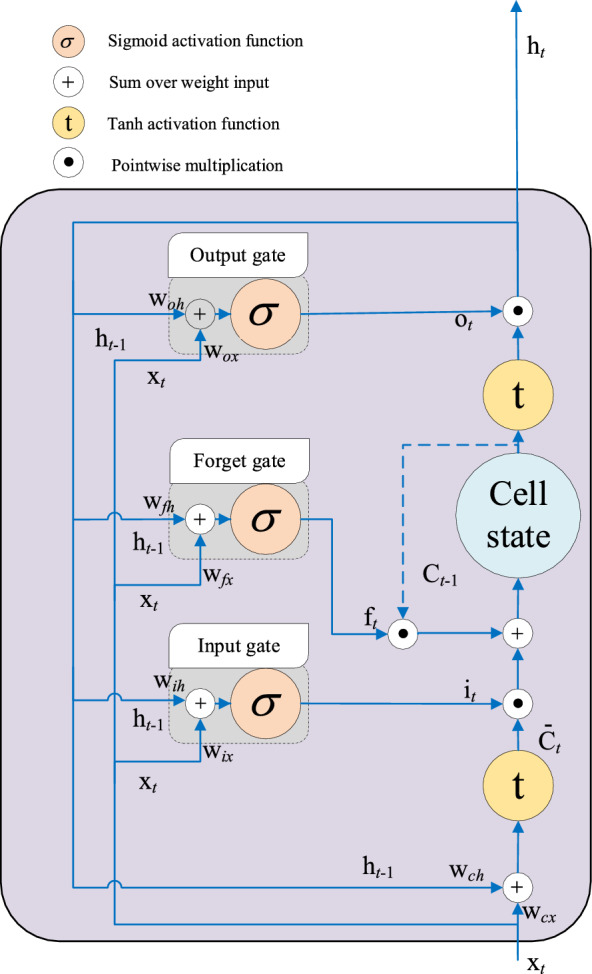
The diagram of LSTM neuron structure.

LSTM^13^ is proposed for releasing the limitation by the nonlinear procession of the data based on the gate mechanism as sown in Fig. 2. The mathematical expression of LSTM is as follows:

7 $$ {\mathbf{i}}_{t} = \sigma \left( {{\mathbf{w}}_{ix} {\mathbf{x}}_{t} + {\mathbf{w}}_{ih} {\mathbf{h}}_{t - 1} + {\mathbf{b}}_{i} } \right) $$

8 $$ {\mathbf{f}}_{t} = \sigma \left( {{\mathbf{w}}_{fx} {\mathbf{x}}_{t} + {\mathbf{w}}_{fh} {\mathbf{h}}_{t - 1} + {\mathbf{b}}_{f} } \right) $$

9 $$ {\mathbf{o}}_{t} = \sigma \left( {{\mathbf{w}}_{ox} {\mathbf{x}}_{t} + {\mathbf{w}}_{oh} {\mathbf{h}}_{t - 1} + {\mathbf{b}}_{o} } \right) $$

10 $$ {\overline{\mathbf{c}}}_{t} = \tanh \left( {{\mathbf{w}}_{cx} {\mathbf{x}}_{t} + {\mathbf{w}}_{ch} {\mathbf{h}}_{t - 1} + {\mathbf{b}}_{c} } \right) $$

11 $$ {\mathbf{c}}_{t} = {\mathbf{f}}_{t} \odot {\mathbf{c}}_{t - 1} + {\mathbf{i}}_{t} \odot {\overline{\mathbf{c}}}_{t} $$

12 $$ {\mathbf{h}}_{t} = {\mathbf{o}}_{t} \odot \tanh \left( {{\mathbf{c}}_{t} } \right) $$

In Eqs. (7–12), the input weight matrixes ${\mathbf{w}}_{ix}$(${\mathbf{w}}_{fx}$, ${\mathbf{w}}_{ox}$, ${\mathbf{w}}_{cx}$) and the recurrent weight matrixes ${\mathbf{w}}_{ih}$(${\mathbf{w}}_{fh}$, ${\mathbf{w}}_{oh}$, ${\mathbf{w}}_{ch}$) are defined by the nonlinear transformation of ${\mathbf{x}}_{t}$ and ${\mathbf{h}}_{t - 1}$ based on forget (input, output) gate, which decides the forget (input, output) degree of data in the hidden layer; ${\mathbf{b}}_{i}$(${\mathbf{b}}_{f}$, ${\mathbf{b}}_{o}$ and ${\mathbf{b}}_{c}$) are the bias of the hidden layer.${\overline{\mathbf{c}}}_{t}$ and ${\mathbf{c}}_{t}$ are the internal state and memory state of the cell;$\odot$ denotes the pointwise multiplication. $\sigma$($\tanh$) is the sigmoid (tanh) activation function.

## The proposed methodology

### Attention-guided multi-hierarchy LSTM

ON-LSTM is first proposed in the NLP field to address the hierarchical structure problem, i.e. "characters, words, and phrases" has a different hierarchy and should be learned in different ways. However, for the vibration signal of mechanical equipment, the hierarchy of order information is difficult to give physical meaning. During the training process, ON-LSTM achieves automatic hierarchy by only providing feedback through the error between predicted and actual results, lacking effective guidance and clear physical interpretation in the hierarchical process. Moreover, the ordered information extracted by ON-LSTM exhibits mixed regions, and the features missed in mixed regions may impact the feature extraction capability. Therefore, this study proposes a new attention-guided multi-hierarchy Long Short-Term Memory (AGMLSTM) neural network that further partitions the mixed hierarchies using the attention mechanism, thereby forming an attention-guided multi-hierarchy information structure. The similarity between the elements of input vectors and recurrent vectors with attention labels determines the segmentation point between input hierarchies and historical hierarchies, which is the index of the most similar element with attention labels. This means that attention is to guide the hierarchical segmentation and give physical meaning to the hierarchy of ordered information of vibration data. Simultaneously, the multi-hierarchy partitioning enables neural networks to fully utilize ordered information. Information that is easily retained over a long period is assigned a high attention hierarchy, while information that is easily replaceable is assigned a low attention hierarchy. The mixed information, representing the intermediate attention hierarchy, is further divided into the sub-hierarchies of low intermediate attention, intermediate attention, and high intermediate attention, which respectively represent short-term information, mid-term information, and long-term information. It should be noted that the intermediate hierarchies (low intermediate, intermediate, and high intermediate) will be zero when the high and low attention hierarchy information has no interaction. In this case, the information in the zone will not participate in the neural network's update process.

**Figure 3:**
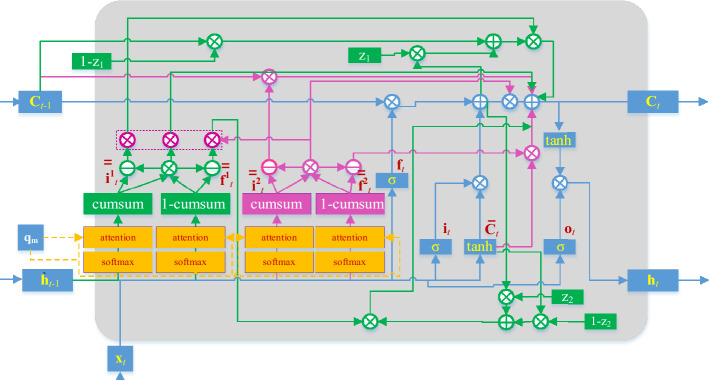
The cellular of AGMLSTM.

Let $${\mathbf{x}}_{t} = \left[ {\begin{array}{*{20}c} {x_{t,1} } & {x_{t,2} } & \ldots & {x_{t,n} } \\ \end{array} } \right]^{T}$$ and $${\mathbf{h}}_{t - 1} = \left[ {\begin{array}{*{20}c} {h_{t - 1,1} } & {h_{t - 1,2} } & \ldots & {h_{{{\text{t - }}1,{\text{m}}}} } \\ \end{array} } \right]^{T}$$ denote the input HIs at time step $t$ and the recurrent data at time step $t - 1$ , respectively. Compared to other networks, the main difference of AGMLSTM lies in the hierarchical information partitioning during the cell unit update process, as illustrated in Fig. 3. The proposed AGMLSTM utilizes attention-guided multi-hierarchy partitioning influenced by attention labels.

By calculating the similarity between input data, recurrent data, and the attention label, the maximum attention coefficient element is identified as the hierarchy segmentation point, so that the model identifies the hierarchy from the largest element to the element that is most similar to the label. Thus, the designed hierarchical structure can be combined with RNN through the attention hierarchies of information. By employing the designed update rules, information with a lower attention hierarchy is more prone to forgetting, while information with a higher attention hierarchy is preserved for a longer duration.

Due to the construction of multi-hierarchy information, let's assume that the main and auxiliary hierarchical positions of the input information ${\mathbf{x}}_{t}$ are denoted as $d_{{_{t,i} }}^{1}$ and $d_{{_{t,i} }}^{2}$, respectively, while the main and auxiliary hierarchical positions of the historical information ${\mathbf{h}}_{t - 1}$ are denoted as $d_{{_{t,f} }}^{1}$ and $d_{{_{t,f} }}^{2}$. These positions are generated using the following construction functions: $F_{1}$, $F_{2}$, $F_{3}$, and $F_{4}$, guided by the query vector $q_{{_{m} }}$. The auxiliary hierarchical positions are used to refine the interval of hierarchical mixing.

13 $$ d_{{_{t,i} }}^{1} = F_{1} {(}{\mathbf{x}}_{t} ,{\mathbf{h}}_{t - 1} ,q_{{{{m}} }} {)} $$

14 $$ d_{t,i}^{2} = F_{2} {(}{\mathbf{x}}_{t} ,{\mathbf{h}}_{{t - {1}}} ,q_{{{m} }} {)} $$

15 $$ d_{t,f}^{1} = F_{3} {(}{\mathbf{x}}_{t} ,{\mathbf{h}}_{{t{ - 1}}} ,q_{{{{m}} }} {)} $$

16 $$ d_{t,f}^{2} = F_{4} {(}{\mathbf{x}}_{t} ,{\mathbf{h}}_{{t - {1}}} ,q_{{{m} }} {)} $$

The memory cell state vector is updated according to certain rules based on the attention hierarchy of input information and recurrent information.

**Figure 4:**
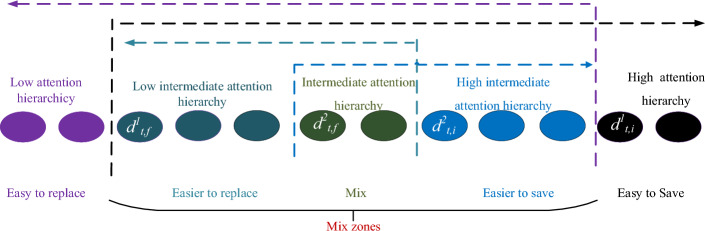
The hierarchy division of AGMLSTM when $d_{{_{t,f} }}^{1} \le d_{{_{t,f} }}^{2} \le d_{{_{t,i} }}^{2} \le d_{{_{t,i} }}^{1}$.

1) If $d_{{_{t,f} }}^{1} \le d_{{_{t,i} }}^{1}$, the main hierarchy of the input information ${\mathbf{x}}_{t}$ is higher than the main hierarchy of the historical information ${\mathbf{h}}_{t - 1}$, resulting in an intermediate attention hierarchy. AGMLSTM is capable of further refining the intermediate attention hierarchy and dividing it into sub-hierarchies: low intermediate attention hierarchy, intermediate attention hierarchy, and high intermediate attention hierarchy, shown in Fig. 4. Therefore, when the hierarchical relationship simultaneously satisfies $d_{{_{t,f} }}^{2} \le d_{{_{t,i} }}^{2}$, the auxiliary hierarchy of the input information ${\mathbf{x}}_{t}$ is also higher than the auxiliary hierarchy of the historical information ${\mathbf{h}}_{t - 1}$. There is an interactive space between $d_{{_{t,f} }}^{2}$ and $d_{{_{t,i} }}^{2}$. The cell unit update rules are as follows: within the cell unit interval $\left[ {{0, \, }d_{{_{t,f} }}^{1} } \right)$, the candidate memory cell state vector ${\overline{\mathbf{c}}}_{t}$ is directly input into the corresponding memory cell, while within the cell unit interval $\left[ {d_{t,i}^{1} ,d_{max} } \right]$, the memory cell state vector ${\mathbf{c}}_{t - 1}$ from the previous time step is directly input into the corresponding memory cell. As for the overlapping region $\left[ {d_{{_{t,f} }}^{1} ,d_{{_{t,i} }}^{1} } \right)$, further refinement updates are performed based on the auxiliary hierarchical positions of the input and historical information. For the overlapping region $\left[ {d_{{_{t,f} }}^{1} ,d_{{_{t,f} }}^{2} } \right)$, the update of ${\mathbf{c}}_{t}$ is:

17 $$ {\mathbf{c}}_{t} { = }{\mathbf{s}}_{1} \odot \left( {{\mathbf{f}}_{t} \odot {\mathbf{c}}_{t - 1} + {\mathbf{i}}_{t} \odot {\overline{\mathbf{c}}}_{t} } \right) + (1 - {\mathbf{s}}_{1} ) \odot {\overline{\mathbf{c}}}_{t} $$

where ${1 - }{\mathbf{s}}_{1}$ is the scale of short-term information in the cellular memory at the case.

For the overlapping region $\left[ {d_{{_{t,f} }}^{2} ,d_{{_{t,i} }}^{2} } \right)$, the update rule of ${\mathbf{c}}_{t}$ is defined as follows:

18 $$ {\mathbf{c}}_{t} { = }\left( {{\mathbf{f}}_{t} \odot {\mathbf{c}}_{t - 1} + {\mathbf{i}}_{t} \odot {\overline{\mathbf{c}}}_{t} } \right) \, $$

For the overlapping region $\left[ {d_{{_{t,i} }}^{2} ,d_{{_{t,i} }}^{1} } \right)$,${\mathbf{c}}_{t}$ is updated by,

19 $$ {\mathbf{c}}_{t} { = }{\mathbf{s}}_{2} \odot \left( {{\mathbf{f}}_{t} \odot {\mathbf{c}}_{t - 1} + {\mathbf{i}}_{t} \odot {\tilde{\mathbf{c}}}_{t} } \right) + (1 - {\mathbf{s}}_{2} ) \odot {\mathbf{c}}_{t - 1} $$

where ${1 - }{\mathbf{s}}_{{2}}$ represents the long-term data ratio. Therefore, under this hierarchy distribution ${\mathbf{c}}_{t}$ is presented bellows,

20 $$ {\mathbf{c}}_{t} = \left( \begin{gathered} \, {\mathbf{c}}_{t - 1} \, \left[ {d_{t,i}^{1} ,d_{max} } \right] \hfill \\ {\mathbf{s}}_{2} \odot \left( {{\mathbf{f}}_{t} \odot {\mathbf{c}}_{t - 1} + {\mathbf{i}}_{t} \odot {\overline{\mathbf{c}}}_{t} } \right) + (1 - {\mathbf{s}}_{2} ) \odot {\mathbf{c}}_{t - 1} \, \left[ {d_{{_{t,i} }}^{2} ,d_{{_{t,i} }}^{1} } \right) \hfill \\ \left( {{\mathbf{f}}_{t} \odot {\mathbf{c}}_{t - 1} + {\mathbf{i}}_{t} \odot {\overline{\mathbf{c}}}_{t} } \right) \, \left[ {d_{{_{t,f} }}^{2} ,d_{{_{t,i} }}^{2} } \right) \hfill \\ {\mathbf{s}}_{1} \odot \left( {{\mathbf{f}}_{t} \odot {\mathbf{c}}_{t - 1} + {\mathbf{i}}_{t} \odot {\tilde{\mathbf{c}}}_{t} } \right) + (1 - {\mathbf{s}}_{1} ) \odot {\overline{\mathbf{c}}}_{t} \, \left[ {d_{{_{t,f} }}^{1} ,d_{{_{t,f} }}^{2} } \right) \hfill \\ \, {\overline{\mathbf{c}}}_{t} \, \left[ {{0},d_{{_{t,f} }}^{1} } \right) \, \hfill \\ \end{gathered} \right) $$

**Figure 5:**
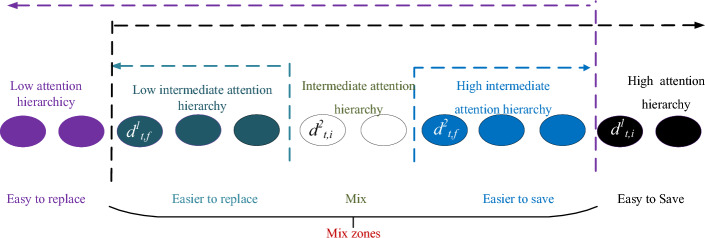
The hierarchy division of AGMLSTM when $d_{{_{t,f} }}^{1} \le d_{{_{t,i} }}^{2} \le d_{{_{t,f} }}^{2} \le d_{{_{t,i} }}^{1}$.

When the hierarchical relationship simultaneously satisfies $d_{{_{t,f} }}^{2} \ge d_{{_{t,i} }}^{2}$, and the auxiliary hierarchical level of the input information ${\mathbf{x}}_{t}$ is lower than the auxiliary hierarchical level of the historical information ${\mathbf{h}}_{t - 1}$, there is no interactive space between $d_{{_{t,f} }}^{2}$ and $d_{{_{t,i} }}^{2}$,, shown in Fig. 5. In this case, the update mechanisms within the index ranges $\left[ {d_{t,i}^{1} ,d_{max} } \right]$ and $\left[ {{0,}d_{{_{t,f} }}^{1} } \right)$ remain consistent with the first case. However, within the index range $\left[ {d_{{_{t,f} }}^{1} ,d_{{_{t,i} }}^{2} } \right)$, the update of ${\mathbf{c}}_{t}$ is as follows:

21 $$ {\mathbf{c}}_{t} { = }{\mathbf{s}}_{{\mathbf{1}}} \odot \left( {{\mathbf{f}}_{t} \odot {\mathbf{c}}_{t - 1} + {\mathbf{i}}_{t} \odot {\overline{\mathbf{c}}}_{t} } \right) + (1 - {\mathbf{s}}_{{\mathbf{1}}} ) \odot {\overline{\mathbf{c}}}_{t} $$

where ${1 - }{\mathbf{s}}_{{\mathbf{1}}}$ is the short-term information ratio. For elements in the range $\left[ {d_{{_{t,i} }}^{2} ,d_{{_{t,f} }}^{2} } \right)$, the cell memory state ${\mathbf{c}}_{t}$ is zero, while in the range $\left[ {d_{{_{t,f} }}^{2} ,d_{{_{t,i} }}^{1} } \right)$ ${\mathbf{c}}_{t}$ is calculated as below,

22 $$ {\mathbf{c}}_{t} { = }{\mathbf{s}}_{2} \odot \left( {{\mathbf{f}}_{t} \odot {\mathbf{c}}_{t - 1} + {\mathbf{i}}_{t} \odot {\overline{\mathbf{c}}}_{t} } \right) + (1 - {\mathbf{s}}_{2} ) \odot {\mathbf{c}}_{t - 1} $$

where ${1 - }{\mathbf{s}}_{{2}}$ denotes the long-term information ratio. In summary, ${\mathbf{c}}_{t}$ at the hierarchy is updated by the below rules,

23 $$ {\mathbf{c}}_{t} = \left( \begin{gathered} \, {\mathbf{c}}_{t - 1} \, \left[ {d_{t,i}^{1} ,d_{max} } \right] \hfill \\ {\mathbf{s}}_{2} \odot \left( {{\mathbf{f}}_{t} \odot {\mathbf{c}}_{t - 1} + {\mathbf{i}}_{t} \odot {\overline{\mathbf{c}}}_{t} } \right) + (1 - {\mathbf{s}}_{2} ) \odot {\mathbf{c}}_{t - 1} \, \left[ {d_{{_{t,f} }}^{2} ,d_{{_{t,i} }}^{1} } \right) \hfill \\ { 0 }\left[ {d_{{_{t,i} }}^{2} ,d_{{_{t,f} }}^{2} } \right) \hfill \\ {\mathbf{s}}_{1} \odot \left( {{\mathbf{f}}_{t} \odot {\mathbf{c}}_{t - 1} + {\mathbf{i}}_{t} \odot {\overline{\mathbf{c}}}_{t} } \right) + (1 - {\mathbf{s}}_{1} ) \odot {\overline{\mathbf{c}}}_{t} \, \left[ {d_{{_{t,f} }}^{1} ,d_{{_{t,i} }}^{2} } \right) \hfill \\ \, {\overline{\mathbf{c}}}_{t} \, \left[ {{0,}d_{{_{t,f} }}^{1} } \right) \hfill \\ \end{gathered} \right) $$

**Figure 6:**
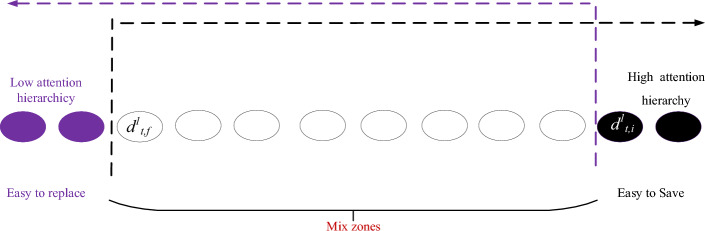
The hierarchy division of AGMLSTM when $d_{{_{t,f} }}^{1} \le d_{{_{t,i} }}^{1}$.

2) If $d_{{_{t,f} }}^{1} \le d_{{_{t,i} }}^{1}$, the main hierarchical level of the input information ${\mathbf{x}}_{t}$ is higher than the main hierarchical level of the historical information ${\mathbf{h}}_{t - 1}$, indicating that the attention focus on the input data than the recurrent data, there are no overlapping cell unit regions. Therefore, ${\mathbf{c}}_{t}$ within the intermediate attention level, there is no need for the mixing of short-term and mid-term memory ${\mathbf{f}}_{t} \odot {\mathbf{c}}_{t - 1} + i_{t} \odot {\overline{\mathbf{c}}}_{t}$ to update. Within the cell unit interval $\left[ {d_{t,i} ,d_{t,f} } \right)$, the current time step's cell activation vector is set to zero. ${\overline{\mathbf{c}}}_{t}$ is the direct input within the cell unit interval $\left[ {{0,}d_{t,i} } \right)$, and for ${\mathbf{c}}_{t - 1}$ is the interval $\left[ {d_{t,f} ,d_{max} } \right]$. At the situation, ${\mathbf{c}}_{t}$ is updated by Eq. (24) with its hierarchical partition shown in Fig. 6.

24 $$ {\mathbf{c}}_{{t^{\prime}}} = \left( {\begin{array}{*{20}c} {{\overline{\mathbf{c}}}_{t} , < d_{t,i} } \\ {{\mathbf{0}}, \, \left[ {d_{t,i} ,d_{t,f} } \right)} \\ {{\mathbf{c}}_{t - 1} , \, \ge d_{t,f} } \\ \end{array} } \right) $$

The construction functions $F_{1}$, $F_{2}$, $F_{3}$ and $F_{4}$ are derived as follows. We first normalize the input data ${\mathbf{x}}_{t}$ and historical data ${\mathbf{h}}_{t - 1}$ using softmax function, introducing four *m*-dimensional vectors ${\overline{\mathbf{f}}}_{t}^{1}$, ${\overline{\mathbf{i}}}_{t}^{1}$, ${\overline{\mathbf{f}}}_{t}^{2}$ and ${\overline{\mathbf{i}}}_{t}^{2}$.

25 $$ {\overline{\mathbf{f}}}_{t}^{1} { = }{\text{softmax}} \left( {{\mathbf{w}}_{{f_{1} }} {\mathbf{x}}_{t} + {\mathbf{w}}_{{f_{1} }} {\mathbf{h}}_{t - 1} + {\mathbf{b}}_{{f_{1} }} } \right) $$

26 $$ {\overline{\mathbf{i}}}_{t}^{1} { = }{\text{softmax}} \left( {{\mathbf{w}}_{{i_{1} }} {\mathbf{x}}_{t} + {\mathbf{w}}_{{i_{1} }} {\mathbf{h}}_{t - 1} + {\mathbf{b}}_{{_{{i_{1} }} }} } \right) $$

27 $$ {\overline{\mathbf{f}}}_{t}^{2} { = }{\text{softmax}} \left( {{\mathbf{w}}_{{f_{2} }} {\mathbf{x}}_{t} + {\mathbf{w}}_{{f_{2} }} {\mathbf{h}}_{t - 1} + {\mathbf{b}}_{{f_{2} }} } \right) $$

28 $$ {\overline{\mathbf{i}}}_{t}^{2} { = }{\text{softmax}} \left( {{\mathbf{w}}_{{i_{2} }} {\mathbf{x}}_{t} + {\mathbf{w}}_{{i_{2} }} {\mathbf{h}}_{t - 1} + {\mathbf{b}}_{{_{{i_{2} }} }} } \right) $$

where ${\mathbf{w}}_{f}$ and ${\mathbf{w}}_{i}$ represent the weight matrices of the softmax layers for historical data and input data, respectively, while ${\mathbf{b}}_{f}$ and ${\mathbf{b}}_{i}$ represent the thresholds of the softmax layers for historical data and input data.

Next, the attention coefficients $\alpha_{{_{t,i} }}^{1}$, $\alpha_{{_{t,i} }}^{2}$, $\lambda_{{_{t,i} }}^{1}$ and $\lambda_{{_{t,i} }}^{2}$ for the input data, and recurrent data are calculated using Eqs. (29–32), respectively:

29 $$ \alpha_{{_{t,i} }}^{1} = \frac{{\exp \left( {s\left( {\overline{i}_{{_{t,i} }}^{1} ,q_{t,m} } \right)} \right)}}{{\sum\limits_{j = 1}^{m} {\exp \left( {s\left( {\overline{i}_{{_{t,j} }}^{1} ,q_{t,m} } \right)} \right)} }} $$

30 $$ \lambda_{{_{t,i} }}^{1} = \frac{{\exp \left( {s\left( {\overline{f}_{{_{t,i} }}^{1} ,q_{t,m} } \right)} \right)}}{{\sum\limits_{j = 1}^{m} {\exp \left( {s\left( {\overline{f}_{{_{t,j} }}^{1} ,q_{t,m} } \right)} \right)} }} $$

31 $$ \alpha_{{_{t,i} }}^{2} = \frac{{\exp \left( {s\left( {\overline{i}_{{_{t,i} }}^{2} ,q_{t,m} } \right)} \right)}}{{\sum\limits_{j = 1}^{m} {\exp \left( {s\left( {\overline{i}_{{_{t,j} }}^{2} ,q_{t,m} } \right)} \right)} }} $$

32 $$ \lambda_{{_{t,i} }}^{2} = \frac{{\exp \left( {s\left( {\overline{f}_{{_{t,i} }}^{2} ,q_{t,m} } \right)} \right)}}{{\sum\limits_{j = 1}^{m} {\exp \left( {s\left( {\overline{f}_{{_{t,j} }}^{2} ,q_{t,m} } \right)} \right)} }} $$

During the training process, the query vector $q_{t,m}$ at this time step $t$ is set as $x_{t + 1,n}$, while during the inference process, it is set as $x_{t,n}$.

The four scoring functions ${\text{s}} \left( {\overline{i}_{{_{t,i} }}^{1} ,q_{t,m} } \right)$, ${\text{s}} \left( {\overline{i}_{{_{t,i} }}^{2} ,q_{t,m} } \right)$, $s\left( {\overline{f}_{t,f}^{1} ,q_{t,m} } \right)$ and $s\left( {\overline{f}_{t,f}^{2} ,q_{t,m} } \right)$ are defined as follows:

33 $$ s\left( {\overline{i}_{{{t,i} }}^{1} ,q_{t,m} } \right) = \frac{{\overline{i}_{t,i}^{1 \, T} q_{t,m} }}{\sqrt m } $$

34 $$ s\left( {\overline{f}_{t,i}^{1} ,q_{t,m} } \right) = \frac{{\overline{f}_{t,i}^{1\, T} q_{t,m} }}{\sqrt m } $$

35 $$ s\left( {\overline{i}_{{{t,i} }}^{2} ,q_{t,m} } \right) = \frac{{\overline{i}_{t,i}^{2\, T} q_{t,m} }}{\sqrt m } $$

36 $$ s\left( {\overline{f}_{t,i}^{2} ,q_{t,m} } \right) = \frac{{\overline{f}_{t,i}^{2T} q_{t,m} }}{\sqrt m } $$

The maximum positions of the attention coefficients $d_{{_{t,i} }}^{1}$ $d_{{_{t,i} }}^{2}$ are set as the main and auxiliary hierarchical positions of the input information ${\mathbf{x}}_{t}$; and the maximum positions of the attention coefficients $d_{{_{t,f} }}^{1}$ and $d_{{_{t,f} }}^{2}$ are set as the main and auxiliary hierarchical positions of the historical information ${\mathbf{h}}_{t - 1}$ , respectively:

37 $$ d_{{_{t,i} }}^{1} = {\text{index}} [\max (\alpha_{{_{t,i} }}^{1} )] $$

38 $$ d_{{_{t,f} }}^{1} = {\text{index}} [\max (\lambda_{{_{t,i} }}^{1} )] $$

39 $$ d_{{_{t,i} }}^{2} = {\text{index}} [\max (\alpha_{{_{t,i} }}^{2} )] $$

40 $$ d_{{_{t,f} }}^{2} = {\text{index}} [\max (\lambda_{{_{t,i} }}^{2} )] $$

where index() denotes as the element position extraction function.

To achieve the automatic hierarchical update as described above, the cumulative sum function cumsum() is used to compute the cumulative sums of the attention coefficients, resulting in the main and auxiliary input gates ${\mathbf{\overline{\overline{i}}}}_{t}^{1}$ and ${\mathbf{\overline{\overline{i}}}}_{t}^{2}$, as well as the main and auxiliary forget gates ${\mathbf{\overline{\overline{f}}}}_{t}^{1}$ and ${\mathbf{\overline{\overline{f}}}}_{t}^{2}$, which can be written as follows:

41 $$ {\mathbf{\overline{\overline{i}}}}_{t}^{1} = 1 - {\text{cumsum}} \left( {{{\varvec{\upalpha}}}_{{_{t} }}^{1} } \right) $$

42 $$ {\mathbf{\overline{\overline{f}}}}_{t}^{1} = {\text{cumsum}} \left( {{{\varvec{\uplambda}}}_{{_{t} }}^{1} } \right) $$

43 $$ {\mathbf{\overline{\overline{i}}}}_{t}^{2} = 1 - {\text{cumsum}} \left( {{{\varvec{\upalpha}}}_{{_{t} }}^{2} } \right) $$

44 $$ {\mathbf{\overline{\overline{f}}}}_{t}^{2} = {\text{cumsum}} \left( {{{\varvec{\uplambda}}}_{{{t} }}^{2} } \right) $$

Then, the attention hierarchy structure is partitioned using the following equations:

45 $$ {\mathbf{w}}_{t}^{0} = {{\overline{\overline{\mathbf i}}}}_{t}^{1} \circ {{\overline{\overline{\mathbf f}}}}_{t}^{1} $$

46 $$ {\mathbf{w}}_{t}^{1} = {\mathbf{\overline{\overline{f}}}}_{t}^{1} - {\mathbf{w}}_{t}^{0} $$

47 $$ {\mathbf{w}}_{t}^{2} = {\mathbf{w}}_{t}^{0} \circ \left( {{\mathbf{\overline{\overline{f}}}}_{t}^{2} - {\mathbf{w}}_{t}^{3} } \right) $$

48 $$ {\mathbf{w}}_{t}^{3} = {\mathbf{w}}_{t}^{0} \circ \left( {{\mathbf{\overline{\overline{f}}}}_{t}^{2} \circ {\mathbf{\overline{\overline{i}}}}_{t}^{2} } \right) $$

49 $$ {\mathbf{w}}_{t}^{4} = {\mathbf{w}}_{t}^{0} \circ \left( {{\mathbf{\overline{\overline{i}}}}_{t}^{2} - {\mathbf{w}}_{t}^{3} } \right) $$

50 $$ {\mathbf{w}}_{t}^{5} = {\mathbf{\overline{\overline{i}}}}_{t}^{1} - {\mathbf{w}}_{t}^{1} $$

In the equation,${\mathbf{w}}_{t}^{1}$ , ${\mathbf{w}}_{t}^{2}$,${\mathbf{w}}_{t}^{3}$ , ${\mathbf{w}}_{t}^{4}$ and ${\mathbf{w}}_{t}^{5}$ represent the high attention hierarchy, high intermediate attention hierarchy, intermediate attention hierarchy, low intermediate attention hierarchy, and low attention hierarchy, respectively.

Finally, with the above equations, the propagation equation of AGMLSTM can be written as follows:

51 $$ \left\{ \begin{gathered} {\mathbf{f}}_{t} = \sigma \left( {{\mathbf{W}}_{f} {\mathbf{x}}_{t} + {\mathbf{U}}_{f} {\mathbf{h}}_{t - 1} + {\mathbf{b}}_{f} } \right) \hfill \\ {\mathbf{i}}_{t} = \sigma \left( {{\mathbf{W}}_{i} {\mathbf{x}}_{t} + {\mathbf{U}}_{i} {\mathbf{h}}_{t - 1} + {\mathbf{b}}_{i} } \right) \hfill \\ {\mathbf{o}}_{t} = \sigma \left( {{\mathbf{W}}_{o} {\mathbf{x}}_{t} + {\mathbf{U}}_{o} {\mathbf{h}}_{t - 1} + {\mathbf{b}}_{o} } \right) \hfill \\ {\overline{\mathbf{c}}}_{t} = \tanh \left( {{\mathbf{W}}_{c} {\mathbf{x}}_{t} + {\mathbf{U}}_{c} {\mathbf{h}}_{t - 1} + {\mathbf{b}}_{c} } \right) \hfill \\ {\hat{\mathbf{c}}}_{t} = {\mathbf{f}}_{t} \odot {\mathbf{c}}_{t - 1} + {\mathbf{i}}_{t} \odot {\tilde{\mathbf{c}}}_{t} \hfill \\ {\mathbf{c}}_{t} = {\mathbf{w}}_{t}^{1} \odot {\mathbf{c}}_{t - 1} + {\mathbf{w}}_{t}^{2} \odot \left[ {z_{2} \odot {\hat{\mathbf{c}}}_{t} + \left( {1 - z_{2} } \right) \odot {\mathbf{c}}_{t - 1} } \right] \hfill \\ + {\mathbf{w}}_{t}^{3} \odot {\hat{\mathbf{c}}}_{t} + {\mathbf{w}}_{t}^{4} \odot \left[ {z_{1} \odot {\hat{\mathbf{c}}}_{t} + \left( {1 - z_{1} } \right) \odot {\overline{\mathbf{c}}}_{t} } \right] + {\mathbf{w}}_{t}^{5} \odot {\overline{\mathbf{c}}}_{t} \hfill \\ {\mathbf{h}}_{t} = {\mathbf{o}}_{t} \odot \tanh \left( {{\mathbf{c}}_{t} } \right) \hfill \\ \end{gathered} \right. $$

where other parameters are the same as LSTM.

### RUL prediction approach

**Figure 7:**
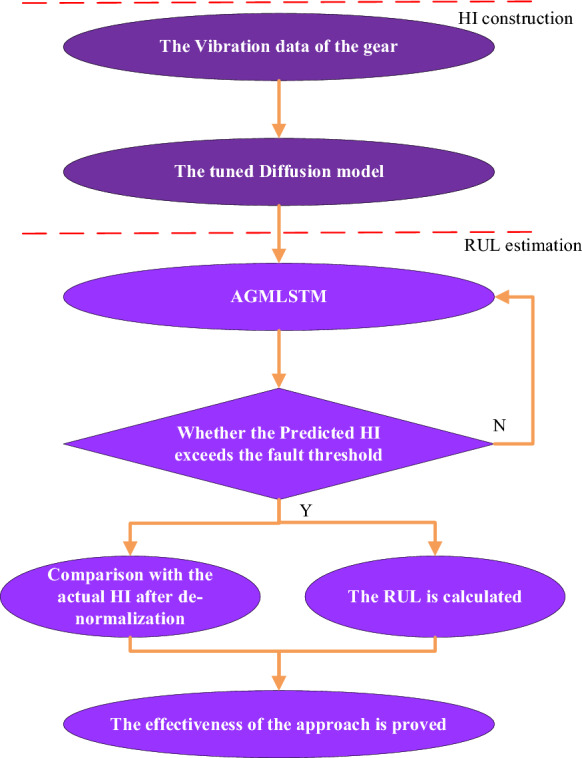
The flowchart of the proposed RUL prediction approach.

A health indicator (HI) that can accurately show the degradation process of gears is crucial to the performance of the prediction model. Therefore, the HI of the vibration signal obtained by the trained diffusion model is used in the article for gear RUL prediction, whose superiority has been demonstrated. Considering that most DL approaches for gear RUL prediction are pattern recognition methods, which are influenced by the quantity and quality of data, an RUL prediction approach under limited samples^20^ is used in the article, whose flowchart is shown in Fig. 7 and the details are presented as follows:

1. The HI data $${\mathbf{z}} = \left[ {\begin{array}{*{20}c} {\begin{array}{*{20}c} {\begin{array}{*{20}c} {z_{1} } & {z_{2} } \\ \end{array} } & \ldots & {z_{n - 1} } \\ \end{array} } & {z_{n} } \\ \end{array} } \right]$$ is calculated based on the full-lifecycle vibration data by the sampling approach whose sampling time is $T$ and sample interval is $\Delta t$.

2. Then the first part $${\mathbf{z^{\prime}}} = \left[ {\begin{array}{*{20}c} {\begin{array}{*{20}c} {\begin{array}{*{20}c} {z_{1} } & {z_{2} } \\ \end{array} } & \ldots & {z_{m - 1} } \\ \end{array} } & {z_{m} } \\ \end{array} } \right]$$ of ${\mathbf{z}}$ is chosen and linearly normalized to obtain $${\mathbf{V}} = \left[ {\begin{array}{*{20}c} {\begin{array}{*{20}c} {\begin{array}{*{20}c} {v_{1} } & {v_{2} } \\ \end{array} } & \ldots & {v_{m - 1} } \\ \end{array} } & {v_{m} } \\ \end{array} } \right]$$.

3. Training pair, containing the model input $$\left[ {\begin{array}{*{20}c} {\begin{array}{*{20}c} {\begin{array}{*{20}c} {{\mathbf{G}}_{1} } & {{\mathbf{G}}_{2} } \\ \end{array} } & \ldots & {{\mathbf{G}}_{l - 1} } \\ \end{array} } & {{\mathbf{G}}_{l} } \\ \end{array} } \right]^{\rm T}$$ and output ${\mathbf{G}}_{{l{ + 1}}}$, is reconstructed by:

52 $$ {\mathbf{G}}{ = }\left[ {\begin{array}{*{20}c} {v_{1} } & {v_{2} } & \cdots & {v_{m - l} } \\ {v_{2} } & {v_{3} } & \cdots & {v_{m - l + 1} } \\ \vdots & \vdots & \ddots & \vdots \\ {v_{l + 1} } & {v_{l + 2} } & \cdots & {v_{m} } \\ \end{array} } \right]{ = }\left[ {\begin{array}{*{20}c} {{\mathbf{G}}_{1} } \\ {{\mathbf{G}}_{2} } \\ \vdots \\ {{\mathbf{G}}_{l + 1} } \\ \end{array} } \right] $$

where the value of *l* is equal to the neural numbers of the input layer and ${\mathbf{G}}_{i}$ is denoted by:

53 $$ {\mathbf{G}}_{i} = \left[ {\begin{array}{*{20}c} {v_{i} } & {v_{i + 1} } & \cdots & {v_{m - l + i - 1} } \\ \end{array} } \right] $$

4. The training loss $L$ of the proposed model is denoted as the mean square error (MSE) between the last row ${\mathbf{G}}_{{{\text{l}} + 1}}$ and the predicted ${\hat{\mathbf{G}}}_{l + 1}$ based on the first *l* rows of the matrix ${\mathbf{G}}$.

54 $$ {\mathbf{y}}_{t} { = }{\hat{\mathbf{G}}}_{l + 1} = f\left( {{\mathbf{G}}_{{\mathbf{1}}} ,{\mathbf{G}}_{2} \cdots ,{\mathbf{G}}_{l} } \right) $$

55 $$ \min \left[ {L\left( {{\mathbf{w}},{\mathbf{b}},{\mathbf{s}}} \right)} \right] = \frac{1}{2}\left[ {{\hat{\mathbf{G}}}_{l + 1} - {\mathbf{G}}_{{{\text{l}} + 1}} } \right]^{2} $$

where *f* denotes the model transaction function; **w**, **b,** and **s** separately denote the learning matrix.

5. After the trained proposed method is obtained, the last *l* is set as the model input to estimate the HI in the next point. Then the step-by-step prediction is executed by:

56 $$ \begin{gathered} {\mathbf{G}}_{l + 2} = f\left( {{\mathbf{G}}_{2} ,{\mathbf{G}}_{3} \cdots ,{\mathbf{G}}_{l + 1} } \right) \hfill \\ {\mathbf{G}}_{l + 3} = f\left( {{\mathbf{G}}_{3} ,{\mathbf{G}}_{4} \cdots ,{\mathbf{G}}_{l + 1} ,f\left( {{\mathbf{G}}_{2} ,{\mathbf{G}}_{3} \cdots ,{\mathbf{G}}_{l + 1} } \right)} \right) \hfill \\ \, \vdots \hfill \\ {\mathbf{G}}_{n} = f\left( {{\mathbf{G}}_{n - l} ,{\mathbf{G}}_{n - l + 1} \cdots ,{\mathbf{G}}_{n - 2} ,f\left( {{\mathbf{G}}_{l - k - 1} ,{\mathbf{G}}_{n - l} \cdots ,{\mathbf{G}}_{n - 2} } \right)} \right) \hfill \\ \end{gathered} $$

6. At last, once the failure threshold is lower than the inversely normalized predicted HIs, the estimated RUL $\overline{Rul}$ is finally obtained by Eq. (57):

57 $$ \overline{Rul} { = }n_{{1}} \times \Delta t $$

where $n_{{1}}$ is the number of predicted HI points before exceeding the threshold. And the actual RUL shows the effectiveness of the proposed method.

### Model optimization

The configuration exploration of the predictive model is executed based on grid search. The hyper-parameters, namely, candidates of learning rate $\alpha$ and neuron number in each layer, are constructed as each grid note, which is searched for optimal predictive performance parameters.

The weight matrix ${\mathbf{w}}$, the bias matrix ${\mathbf{b}}$, and the proportion matrix ${\mathbf{s}}$ of the model are trained during the training stage based on the loss function Eq. (55) and updated on Eq. (58) by Adam optimizer.

58 $$ \left[ {\begin{array}{*{20}c} {{\mathbf{w}}_{t + 1}^{{}} } \\ \begin{gathered} {\mathbf{b}}_{t + 1} \hfill \\ {\mathbf{s}}_{t + 1}^{{}} \hfill \\ \end{gathered} \\ \end{array} } \right] = \left[ {\begin{array}{*{20}c} {{\mathbf{w}}_{t}^{{}} } \\ \begin{gathered} {\mathbf{b}}_{t} \hfill \\ {\mathbf{s}}_{t}^{{}} \hfill \\ \end{gathered} \\ \end{array} } \right] - \alpha \left[ {\begin{array}{*{20}c} {\frac{{\partial L_{t} }}{{\partial {\mathbf{w}}_{t}^{{}} }}} \\ \begin{gathered} \frac{{\partial L_{t} }}{{\partial {\mathbf{b}}_{t}^{{}} }} \hfill \\ \frac{{\partial L_{t} }}{{\partial {\mathbf{s}}_{t}^{{}} }} \hfill \\ \end{gathered} \\ \end{array} } \right] $$

## Experimental analysis

Several fatigue full-life experiments are executed by a gear contact fatigue test rig to investigate the lifespan of gears from normal conditions to failure (tooth broken and pitting). The material of the gear for the tooth fracture case was 40Cr, while the gear material for the pitting case was 20CrMnTi. The gear module was set to 5, and the experimental gear case had an oil flow rate of 4 L/h with a cooling temperature of 70 °C. The gears that experienced tooth-broken failures (Dataset 1 and Dataset 2) had tooth counts of 31, 25, 25, and 31, respectively. On the other hand, the gears that suffered from pitting failures (Dataset 3 and Dataset 4) had tooth counts of 26, 24, 24, and 26, as shown in Table 1.

**Table 1:** Description of data.

Dataset	Data 1	Data 2	Data 3	Data 4
Load (KN)	1.4	1.4	1.2	1.2
Speed (rpm)	500	500	1000	1000
Test time (min)	814	820	696	951
Number of samples	814	820	696	951
Failure mode	Broken	Broken	Pitting	Pitting

**Figure 8:**
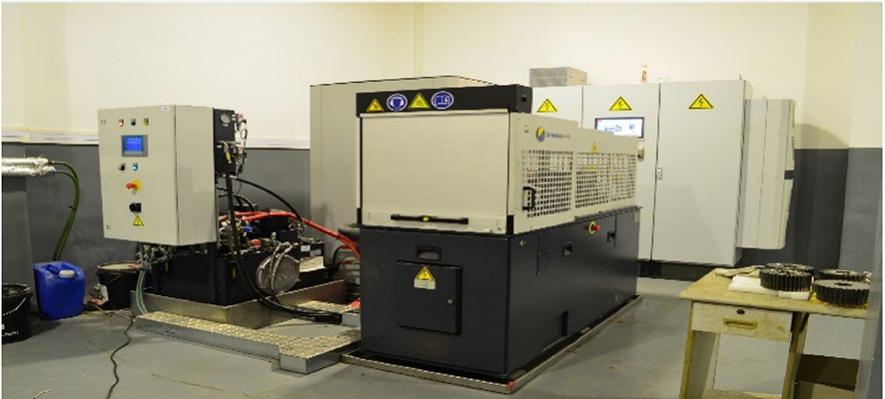
Gear contact fatigue testing machine.

As depicted in Fig. 8, the experimental setup comprises a torque controller, a cooling and lubrication controller, an experimental operation platform, and a gear operation platform. The sampling frequency for the experimental setup is fixed at 50,000 Hz. To minimize data volume, this study sets the recording interval, and the sampling length are 60 s and 10 s. And Part of the healthy state data at the beginning of the run is deleted. Data sets 1 and 3 are used to train the Diffusion model for calculating gear HIs. Then, the trained Diffusion model is used to encode the health indicator points of data sets 2 and 4. To test the prediction ability of the predictive model, this study conducts experiments using the health indicator points from all data sets. Through grid search, optimal hyper-parameters for the AGMLSTM are obtained. For data sets 1, 3, and 4, the number of neurons in the input, hidden, and output layers of AGMLSTM are set to 100, 35, and 1. For data set 2, they are set to 60, 20, and 1. The learning rates for the models on data sets 1, 2, 3, and 4 are set to 0.02, 0.03, 0.05, and 0.05.

**Figure 9:**
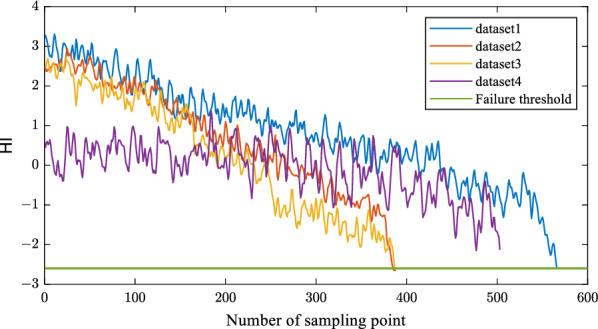
HI of four datasets.

Appropriate health indicators can effectively reflect the health condition of mechanical equipment and improve the RUL prediction capability^26–30^. Due to the limitations of single features such as root mean square, kurtosis, and frequency centroid, they may not adequately capture the degradation trend of mechanical equipment in most data sets. Therefore, this study develops a health indicator based on diffusion model that can be used in most cases. Since the signals collected during the steady-state phase contain less degradation information, only a portion of the samples from the lifecycle data set is used to calculate the health indicator points using diffusion model and then applied to remaining useful life prediction. Figure 9 displays the obtained health indicator points for all four gear data sets. The constructed health indicator point curves can effectively reflect the degradation trend of gear health, which is highly beneficial for RUL prediction. All gear health indicator curves exhibit a declining trend, and their failure thresholds are similar. This aids in setting a unified failure threshold for different experimental setups, thereby enhancing the robustness of gear RUL prediction.

**Figure 10:**
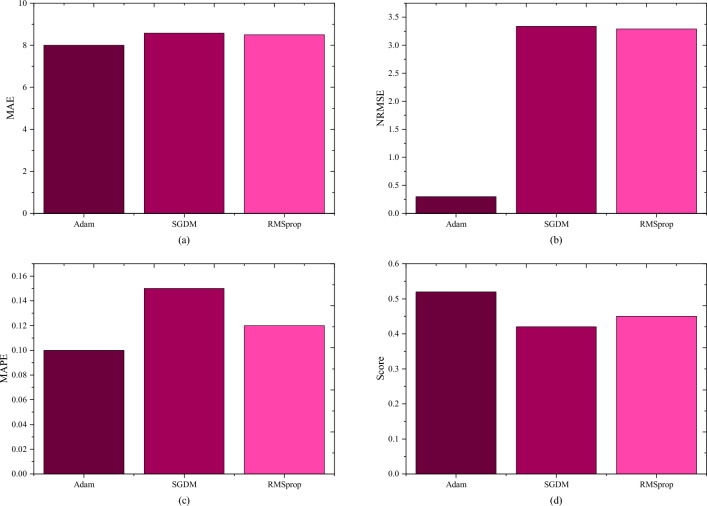
Comparison of predictive ability under different optimizers.

The study undertook comparative experiments employing distinct optimization algorithms to underscore the superior performance of the chosen optimizer. Specifically, SGDM^31^, RMSprop^32^, and Adam were deliberately selected for comparison within a consistent structural framework, and subsequent optimization was applied across all models. The evaluation process involved ten parallel experiments for each model, focusing on a one-hour prediction task. Model performance was rigorously assessed using key performance indicators, namely the mean absolute error (MAE), the normalized root mean square error (NRMSE), the mean absolute percentage error (MAPE), and Score^23^, as presented in Fig. 10.

It can be concluded that the model adopted by Adam has the lowest values of MAE, NRMSE, and MAPE, and the highest Score value. This means that with the Adam optimizer, the proposed method has better RUL prediction performance. Thus, Adam is more suitable for the proposed method when it deals with gear RUL prediction.

The evaluation indicators of different HIs for different gear datasets are respectively calculated and the mean value of evaluation indicators are listed in Table 2. First the two widely used statistical features such as RMSE and Kurtosis in PHM^20^ are chosen as HIs. Then HI based on popularity learning is constructed, i.e. PCA. Finally, HIs are constructed by other Unsupervised networks deep belief network (DBN)^33^, and variational autoencoder (VAE)^24^.

**Table 2:** The evaluation indicators of different HIs for datasets.

	Mon	Corr	Rob	CI
RMSE	0.519	0.706	0.826	0.636
Kurtosis	0.414	0.618	0.737	0.540
PCA	0.754	0.863	0.674	0.771
DBN	0.916	0.802	0.653	0.829
VAE	0.829	0.853	0.830	0.837
Diffusion model	0.955	0.921	0.890	0.932

In Table 2, a comprehensive analysis of the evaluation indicators for HIs reveals that those generated by the diffusion model consistently outperform other HIs across gear datasets. Notably, the values of monotonicity and the comprehensive indicator for the diffusion model-reconstructed HI stand out, reaching impressive scores of 0.955 and 0.921, respectively. This signifies that the HI constructed through the diffusion model adeptly captures and reflects the degradation trend in gear datasets. The comparison across different HIs reveals that those generated by DBN, VAE, and the diffusion model surpass those based on PCA, RMSE, and Kurtosis. This suggests that HIs constructed by neural networks exhibit greater flexibility when dealing with HIs under fixed patterns, although they may not be ideal for reflecting the degradation trend in gear datasets. Besides, the diffusion model stands out by delivering strong performance evaluation results. This highlights its superior generalization ability, indicating that the HIs produced by the diffusion model are well-suited for assessing health status in gear datasets. Consequently, the HIs constructed by the diffusion model effectively and reliably capture the degradation trend in gear systems.

**Figure 11:**
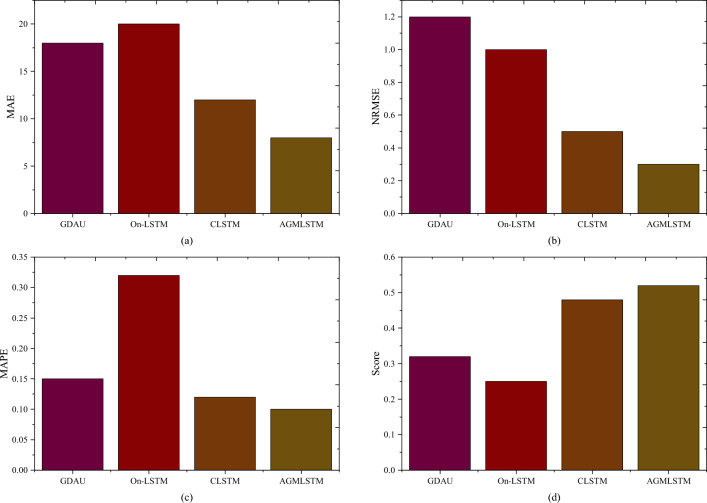
The gear RUL estimation performance of different methods.

Using the small-sample life prediction method, the proposed AGMLSTM is compared with classical models (LSTM, GRU) and published deep learning models, i.e. Gated dual attention unit (GDAU)^20^, On-LSTM^21^, Coctail LSTM (CLSTM)^24^, for RUL prediction on the four gear data sets. To compare the prediction accuracy and robustness of each method, grid search is used to obtain the optimal hyper-parameters for each model, and then all tuned networks are tested 10 times on each gear data set. The prediction task is set as predicting 60 HI points (1-h RUL) for the comparative experiment, comparing the prediction capabilities of the benchmark models. Based on the experimental prediction results, MAE, NRMSE, MAPE and Score are used to quantitatively evaluate the prediction accuracy, as shown in Fig. 11.

As illustrated in Fig. 11, the superiority of the proposed AGMLSTM model over other counterparts is evident, showcasing exceptional performance in predicting RUL. This observation underscores the significant impact of incorporating comprehensive ordered information, especially when employing attention mechanisms at the hidden layer level. The strategic utilization of attention mechanisms facilitates the network models in effectively navigating data heterogeneity, leading to a remarkable enhancement in RUL estimation accuracy. Based on the actual gear tests, the outperformance of the prosed RUL prediction method is proven by MAE, NRMSE, MAPE, and Score, with improvement of 33%, 40%, 17%, and 8% respectively compared with the state-of-art. Consequently, the proposed models emerge as highly apt for the precise prediction of gear remaining useful life, attributing their success to the adept utilization of ordered information and attention-guided learning mechanisms.

AGMLSTM and CLSTM refine the mixed hierarchy through fine-grained processing based on the introduced main and auxiliary gating mechanisms. The distinction lies in the fact that AGMLSTM employs an attention mechanism for hierarchical localization. Consequently, while AGMLSTM and CLSTM achieve better RUL prediction accuracy compared to ON-LSTM and GDAU, they come with an increased parameter count. With the same number of hidden layer neurons $L_{n}$, AGMLSTM increases the parameter count compared to $8 \times L_{n}$ ON-LSTM and $16 \times L_{n}$ GDAU, and is approximately equivalent to CLSTM. To provide a more intuitive representation of the network's computational complexity, we calculated the time required for each iteration during the training process on the same computer device, as shown in Table 3.

**Table 3:** The complexity analysis of models.

	On-LSTM	GDAU	CLSTM	AGMLSTM
Train time in each epoch(s)	0.12	0.15	0.17	0.18

From Table 3, it is evident that AGMLSTM and CLSTM incur a higher time cost than On-LSTM. This is attributed to the different hierarchical learning mechanisms these models employ for input processing, with additional gating units introducing more network parameters. The GDAU, which incorporates dual attention gates, exhibits a similar phenomenon. Additionally, it is crucial to note that the training phase is offline, and during the online prediction phase, the trained AGMLSTM incurs a prediction time of only 7.8 $\times$ 10^–5^ s. Hence, the prediction time overhead of AGMLSTM is deemed acceptable considering its superior long-term RUL prediction accuracy.

Based on the above analysis, the rational and comprehensive use of ordered information is crucial for enhancing the accuracy of gear RUL prediction, especially in cases where known samples contain less gear degradation information. Therefore, the proposed method AGMLSTM, guided by an attention mechanism for multi-hierarchy partitioning, effectively extracts more gear state degradation information, resulting in superior overall RUL prediction performance compared to other methods.

**Figure 12:**
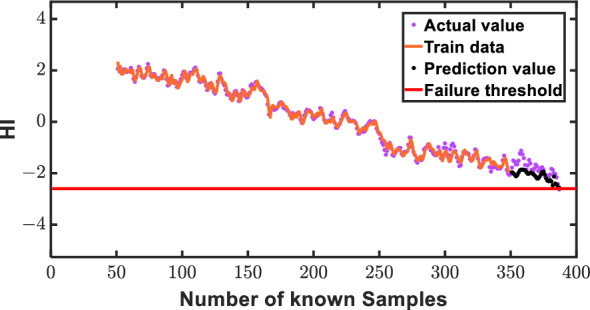
Prediction illustration for 30 predicted points of data 3.

**Figure 13:**
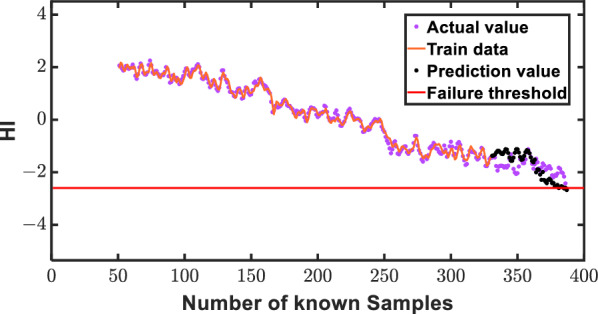
Prediction illustration for 60 predicted points of data 3.

**Figure 14:**
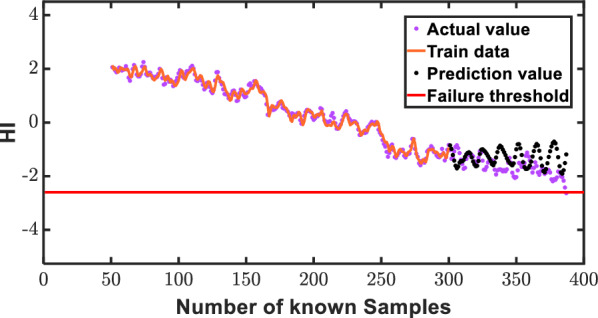
Prediction illustration for 90 predicted points of data 3.

Illustrating the robustness of our proposed small-sample intelligent prediction method, we employ data set 3 as a paradigmatic case study, harnessing the AGMLSTM model for an insightful exploration of RUL prediction across diverse forecast horizons. The delineation of the training set, consisting of known data from the initial segment, and the validation set, featuring unknown data from the subsequent portion, lays the groundwork for a comprehensive evaluation. Intriguingly, the AGMLSTM model's prowess is vividly showcased through an in-depth analysis of its predictive capabilities on data set 1, where the focus is squarely on anticipating 90, 70, and 50 HIs. As delineated in Figs. 12, 13 and 14, a compelling narrative unfolds, elucidating a direct correlation between the increasing number of known HIs and the model's augmentation in prediction proficiency. The figures distinctly reveal a convergence of estimated health indicator points towards their true counterparts, affirming the method's precision and efficacy. Crucially, the overarching alignment between prediction values and actual values across a spectrum of forecast instances underscores the AGMLSTM model's unparalleled effectiveness in gear RUL prediction. This nuanced ability to predict with heightened precision as our understanding of health indicators expands substantiates the model's robustness and underscores its potential for real-world applications.

**Figure 15:**
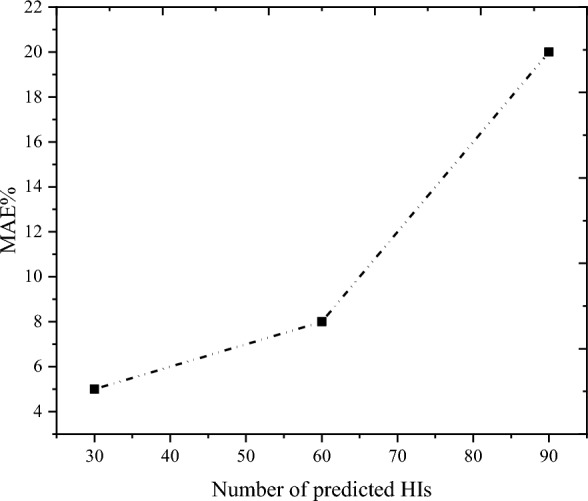
MAEs of RUL prediction results under different known HI points.

In Fig. 15, the prowess of AGMLSTM in predicting RUL at varying known health indicator points is rigorously assessed using the MAE. A compelling trend unfolds, revealing a noteworthy inverse correlation: the MAE values exhibit a consistent decline as the number of health indicator points rises. This observation underscores the model's heightened proficiency with an expanding set of health indicators. Examining specific instances, for a prediction involving 30 health indicator points, the RUL prediction boasts a mere 5% percentage error. Intriguingly, with an escalation to 60 health indicator points, the percentage error marginally increases to 8%. The augmentation of known HIs entails the incorporation of expanding HIs encompassing fault information into the model training process. This influx of HIs allows the model to assimilate a broader spectrum of fault trends, leading to a progressive enhancement in its predictive capabilities. These outcomes signify AGMLSTM's commendable performance in protracted RUL prediction, showcasing its capacity for sustained accuracy. To further underscore the model's prowess in long-term RUL estimation, a bold attempt is made to predict 90 health indicator points, as illustrated in Fig. 14 Despite a 25% error in the computed result, this endeavor unequivocally establishes AGMLSTM's formidable predictive aptitude for enduring gear RUL scenarios.

## Conclusion

Revolutionizing gear RUL prediction, our groundbreaking approach introduces a novel methodology by constructing HIs through a diffusion model, coupled with the innovative AGMLSTM predictor. Leveraging the temporal and frequency characteristics of vibration measurements, the diffusion model lays the foundation for a distinctive gear HI. This HI, in turn, serves as the linchpin for AGMLSTM, a pioneering predictor designed to comprehensively and judiciously mine ordered information for precise gear RUL forecasts. The strategic incorporation of rich ordered information significantly amplifies the feature extraction capabilities of our predictor, leading to a substantial enhancement in RUL prediction accuracy. Validation through rigorous real-world gear tests unequivocally demonstrates the superior performance of our proposed RUL prediction method. Employing widely accepted evaluation metrics, our approach realizes 8 on MAE, 0.3 on NRMSE, 0.1 on MAPE, and 0.52 on Score, showcasing an impressive improvement of 33%, 40%, 17%, and 8% respectively, compared to state-of-the-art methods. In essence, our proposed approach emerges as the pinnacle of gear RUL prediction methodologies, providing not only heightened accuracy but also unparalleled effectiveness in real-world scenarios.

The proposed methodology in this study primarily addresses the RUL under conditions of single-tooth breakage or pitting failure. However, in practical engineering applications, failures frequently involve the coupling of multiple faults. Therefore, the development of a methodology for predicting the RUL in cases of complex gearbox failure is of significant importance. This aspect will be a key focus of our future research endeavors.

## Data Availability

The datasets used and/or analyzed during the current study available from the corresponding author on reasonable request.
